# Constitutional epimutations in *LTBP4*, a component of the TGF-β signaling, and in *BRCA1*, as potential drivers of early-onset colorectal cancer

**DOI:** 10.1186/s13148-025-01924-x

**Published:** 2025-11-05

**Authors:** Mariona Terradas, Pilar Mur, Francisco D. Morón-Duran, Pol Mengod, Chiara M. L. Löffler, Noah C. Helderman, Diantha Terlouw, Xavier Sanjuán, Pablo Bousquets-Muñoz, Julen Viana-Errasti, Xose S. Puente, Gabriel Capellá, Maartje Nielsen, Tom van Wezel, Jakob Nikolas Kather, Conxi Lázaro, Victor Moreno, Laura Valle

**Affiliations:** 1 Hereditary Cancer Program, Catalan Institute of Oncology; Oncobell Program, IDIBELL, Hospitalet de Llobregat, Barcelona, Spain; 2 Centro de Investigación Biomédica en Red de Cáncer (CIBERONC), Madrid, Spain; 3 Unitat de Biologia Cel·lular i Genètica Mèdica, Facultat de Medicina, Universitat Autònoma de Barcelona, Cerdanyola del Vallès, Barcelona, Spain; 4 Department of Health of Catalonia, Catalan Cancer Plan, Barcelona, Spain; 5 Unit of Biomarkers and Susceptibility, Oncology Data Analytics Program (ODAP), Catalan Institute of Oncology, Hospitalet de Llobregat, Barcelona, Spain; 6 Consortium for Biomedical Research in Epidemiology and Public Health (CIBERESP), Madrid, Spain; 7 Department of Clinical Sciences, School of Medicine and Universitat de Barcelona Institute of Complex Systems (UBICS), University of Barcelona, Barcelona, Spain; 8 Else Kroener Fresenius Center for Digital Health, Faculty of Medicine, University Hospital Carl Gustav Carus, TUD Dresden University of Technology, Dresden, Germany; 9 Department of Internal Medicine I, University Hospital Carl Gustav Carus, Dresden, Germany; 10 National Center for Tumor Diseases Dresden (NCT/UCC), a Partnership Between DKFZ, Faculty of Medicine and University Hospital Carl Gustav Carus, TUD Dresden University of Technology, and Helmholtz-Zentrum Dresden - Rossendorf (HZDR), Dresden, Germany; 11 Department of Clinical Genetics, Leiden University Medical Center, Leiden, The Netherlands; 12 Department of Pathology, Leiden University Medical Center, Leiden, The Netherlands; 13 Department of Pathology, Hospitalet de Llobregat, Bellvitge University Hospital, Barcelona, Spain; 14 Departamento de Bioquímica y Biología Molecular, Instituto Universitario de Oncología (IUOPA), Universidad de Oviedo, Oviedo, Spain; 15 Programa de Doctorat en Biomedicina, Hospitalet de Llobregat, Universitat de Barcelona (UB), Barcelona, Spain; 16 Medical Oncology, National Center for Tumor Diseases (NCT), University Hospital Heidelberg, Heidelberg, Germany

**Keywords:** Hereditary cancer, Promoter methylation, Constitutional epimutation, Early-onset colorectal cancer, *LTBP4*, *BRCA1*

## Abstract

**Background:**

Constitutional primary monoallelic promoter methylation of hereditary cancer genes, although rare, may explain early-onset cancers without family history. Also, promoter methylation of a hereditary cancer gene secondary to a genetic alteration in a methylation regulatory region can cause a hereditary cancer syndrome. This study investigates constitutional promoter methylation as mechanism of inactivation of cancer predisposition genes in genetically unsolved familial and/or early-onset colorectal cancer (CRC) patients.

**Results:**

Bisulfite-treated peripheral blood DNA from 46 early-onset/familial CRC patients was analyzed using the Illumina Infinium MethylationEPIC BeadChip. One early-onset CRC patient exhibited constitutional, likely monoallelic, methylation of CpG island 102 in *LTBP4*, a gene involved in TGF-β signaling. Somatic methylation of this CpG island is common in CRC, and correlates with *LTBP4* downregulation. *LTBP4* double knockout mice develop colorectal adenomas and carcinomas, supporting the role of this gene in CRC predisposition. No additional cases with constitutional *LTBP4* CpG island 102 methylation or enrichment of deleterious *LTBP4* variants in CRC patients compared to controls were found. Another early-onset CRC patient exhibited mosaic *BRCA1* promoter methylation, typically associated with increased breast and ovarian cancer risk. No somatic second hit in *BRCA1* was detected in the patient’s tumor, and homologous recombination deficiency-associated features were inconclusive.

**Conclusions:**

Our findings suggest that constitutional methylation of *LTBP4* CpG island 102 may be associated with increased CRC risk. Identification of additional cases is needed to confirm the existence of a novel CRC predisposition syndrome driven by epigenetic inactivation of *LTBP4*, potentially also linked to other clinical phenotypes associated with *LTBP4* deficiency, such as pulmonary emphysema. Whether constitutional *BRCA1* methylation contributes to CRC risk remains to be determined.

**Supplementary Information:**

The online version contains supplementary material available at 10.1186/s13148-025-01924-x.

## Background

Genetic predisposition to nonpolyposis colorectal cancer (CRC) is mainly explained by genetic and epigenetic alterations in the DNA mismatch repair (MMR) genes *MLH1*, *MSH2*, *MSH6,* and *PMS2*, causing Lynch syndrome. Also, germline pathogenic variants in *RPS20* have been causally linked to very few cases of hereditary nonpolyposis MMR-proficient CRC [1]. Besides *RPS20*, the anecdotal identification of germline pathogenic variants in some polyposis genes (*MUTYH*, *POLE*, *POLD1*, *BMPR1A*) in cases with nonpolyposis CRC phenotypes, the low or marginal CRC risks reported for other hereditary cancer genes such as *ATM*, *CHEK2*, *TP53*, and *BRCA1*, or the low-penetrance *APC* variant I1307K [2–6], no other genes have been unequivocally associated with MMR-proficient nonpolyposis CRC predisposition [7].

Constitutional monoallelic inactivation of hereditary cancer genes through promoter methylation is rarely responsible for cancer predisposition. This mechanism has been reported in Lynch syndrome affecting *MSH2* as a consequence of deletions involving the neighboring upstream *EPCAM* gene, and *MLH1*, mainly as primary epimutations—i.e., not associated with a genetic alteration [8]. Constitutional epigenetic alterations have also been described in *BRCA1* and *RAD51*, predisposing to breast and/or ovarian cancer [9–11]; in *DAPK1,* associated with increased risk of chronic lymphocytic leukemia [12]; and in *KILLIN*, identified in Cowden and Cowden-like syndromes in the absence of constitutional *PTEN* mutations [13].

Our aim consisted of assessing the presence of aberrant constitutional methylation in known or new hereditary cancer genes as cause of familial and/or early-onset MMR-proficient CRC. We performed a genome-wide methylation analysis in blood DNA obtained from 46 unrelated patients affected with early-onset CRC and/or with familial aggregation of the disease, and absence of germline pathogenic variants in known hereditary CRC or polyposis genes.

## Methods

**Fig. 1:**
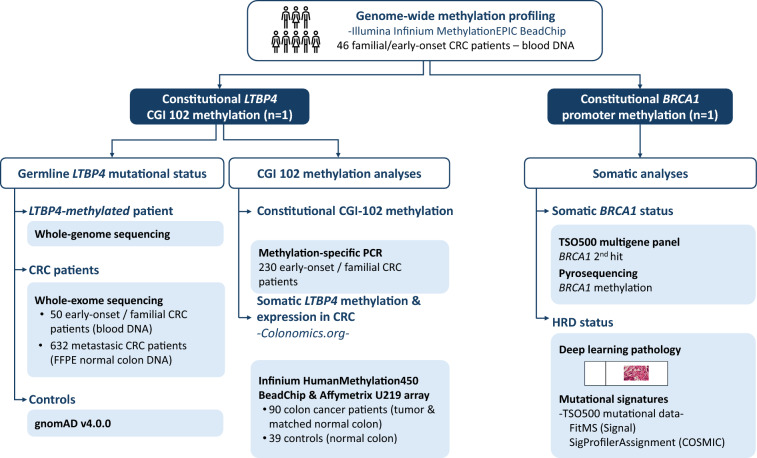
Methodological workflow of the study aimed to identify and characterize constitutional methylation alterations identified in early-onset CRC cases. Abbreviations: CGI, CpG island; CRC, colorectal cancer; HRD, homologous repair deficiency

The methodological workflow of the study is represented in Fig. 1.

### Patients

Genome-wide methylation analysis in blood DNA was performed in 46 MMR-proficient nonpolyposis CRC patients (Suppl. Table 1) recruited through the Hereditary Cancer Program of the Catalan Institute of Oncology (Spain). Patients were selected based on: i) absence of MMR deficiency, assessed by immunohistochemistry and/or microsatellite analysis (100% of cases); ii) absence of germline pathogenic variants in *MUTYH* (biallelic), *NTHL1* (biallelic), *MBD4* (biallelic), *BMPR1A*, or the exonuclease domains of *POLE* and *POLD1* (100% of cases); and iii) early age at CRC diagnosis (45/46 diagnosed before age 50).

*LTBP4* CpG island 102-specific constitutional methylation analysis was performed in 230 MMR-proficient familial and/or early-onset nonpolyposis CRC patients, with no germline pathogenic variants in known hereditary CRC or polyposis genes, also assessed at the Hereditary Cancer Program of the Catalan Institute of Oncology (Spain).

*LTBP4* germline mutational status was evaluated in: (1) 50 familial and/or early-onset genetically unexplained MMR-proficient CRC unrelated patients; and (2) 632 patients diagnosed with metastatic CRC. Fourteen familial/early-onset CRC patients were recruited through the Hereditary Cancer Program of the Catalan Institute of Oncology (Spain), and all the other patients through the Departments of Clinical Genetics and Pathology of Leiden University Medical Center (LUMC, The Netherlands). See Supplementary Material and Methods for additional details.

### Genome-wide methylation profiling

1 µg of blood DNA was treated with bisulfite using the EZ DNA Methylation Gold™ kit (Zymo Research, CA, USA) following the manufacturer’s instructions. Genome-wide methylation analysis was performed with the Infinium MethylationEPIC BeadChip (Illumina, CA, USA), which includes >850,000 CpGs. CpG β-values were obtained using the GenomeStudio software (Illumina). For every individual patient, each β-value was compared to the β-values of the other 45 patients, using the Qlucore Omics Explorer 3.8 (Qlucore, Sweden). Methylation of a CpG region was considered when at least three consecutive CpGs were differentially methylated in a patient (FDR, *q*-value < 0.005).

### Whole-genome sequencing

Whole-genome sequencing (WGS) of blood DNA from patient P-38 was performed using Illumina technology. Libraries were prepared using the TruSeq DNA PCR-Free Library Preparation Kit (Illumina) and sequencing was conducted on a HiSeq X™ Ten Sequencing System (Illumina), with is based on Illumina SBS technology. Image processing and base calling were performed through an integrated primary analysis software called RTA2 (Real Time Analysis 2). The Illumina bcl2fastq2-v2.20.0 conversion software was used to demultiplex sequencing data and convert base call (BCL) into FASTQ files. Genome alignment, variant calling, and identification of single-nucleotide variants (SNVs), and small insertions/deletions (indels) were performed following the same pipeline as for whole-exome sequencing (WES) (Suppl. Material and Methods). Copy number variants and structural variants in the region of interest were assessed using the Integrative Genomics Viewer (IGV) [14].

### Somatic *LTBP4 *CpG methylation in colorectal cancer and paired normal colon mucosa, and correlation of *LTBP4* methylation with *LTBP4* expression in tumors

Information on the status of somatic methylation of *LTBP4* CpGs (Illumina Infinium HumanMethylation450 BeadChip) and gene expression (Affymetrix Human Genome U219 Array) in colon cancer and paired adjacent normal colon mucosa samples, as well as in normal colon mucosa from cancer-free donors was obtained from the Colonomics project (www.colonomics.org) [15]. Colonomics includes methylation and RNA expression data from 90 tumor samples and their paired adjacent normal mucosa, as well as 39 normal colon biopsies from cancer-free individuals.

The relationship between *LTBP4* gene expression and methylation at CpG sites within CpG island 102 (chr19:41,119,032–41,120,394, GRCh37/hg19) was assessed using Pearson correlation coefficients. Statistical significance of the correlations was evaluated  with two-tailed t-tests.

*LTBP4* expression was summarized using the first principal component (PC1) derived from principal component analysis (PCA) of the signal generated by the different probes targeting *LTBP4* on the Affymetrix Human Genome U219 Array Plate platform. Expression data were previously normalized using the Robust Multiarray Average (RMA) algorithm from the Affy package (version 1.28) in R/Bioconductor, and rescaled to reflect the average expression of the probes with the highest observed standard deviation.

*LTBP4* methylation was assessed using probes in the region covering CpG islands on the Illumina Infinium HumanMethylation450 BeadChip. We used β-values calculated with the average probe intensity at each locus ranging from 0 (absence of methylation) to 1 (complete methylation), and technical variability was reduced within and between arrays by subset quantile within array normalization (SWAN) using the Minfi package (version 3.0.1) from R/Bioconductor. The Colonomics data used in this analysis are publicly available at the CORA research data repository (doi:10.34@810/data169) [16].

### Methylation-specific PCR-based sequencing

Methylation-specific PCR-based sequencing, also known as direct bisulfite sequencing, was used for the validation of the Infinium MethylationEPIC array positive results and for the *LTBP4* CpG island 102-specific methylation analysis in 235 additional familial and/or early-onset unsolved CRC patients. Bisulfite-converted DNA was amplified by PCR using NZYTaq II 2 × Colorless mastermix (Nzytech, Portugal) and the following primer pairs (from 5’ to 3’): CpG102.1_FW: GGGAGGAGTTTAGAAATTTGGTAT and CpG102.1_RV: CAAAAACACAAATACAAACAAAAA; CpG102.3_FW: ATTAGGGGGTAGTTGGTGGGAGTTT and CpG102.3_RV: AATTTAAAACAACTTCTTTACAATCAC. Direct automated sequencing was performed at Stabvida (Caparica, Portugal).

### *BRCA1* promoter methylation evaluation by pyrosequencing

Tumor DNA extraction from the FFPE material obtained from a hematoxylin-eosin-stained slide was performed using the QIAamp DNA FFPE Tissue Kit (Qiagen, Hilden, Germany). DNA was treated with bisulfite using the EZ DNA Methylation-DirectTM kit (Zymo Research Corporation, Irvine, CA, USA). Promoter methylation status of *BRCA1* was analyzed using specific forward and reverse primers (one of them biotin 5’-labeled) [17], allowing the analysis of four CpG sites of the *BRCA1* promoter by pyrosequencing. Biotinylated amplicons were conjugated with streptavidin and recovered using the PyroMark Vacuum Prep Workstation (Qiagen). Pyrosequencing was carried out on a PyroMark Q24 instrument (Qiagen), using a target-specific sequencing primer [17]. Analysis was performed using the PyroMark Q24 2.0.8 software (Qiagen). Methylation status of the *BRCA1* promoter was quantified in terms of methylation mean, as the mean percentage of methylated cytosines at four CpG sites.

### *LTBP4* mutational status

*LTBP4* sequencing data were extracted from WES results obtained from blood DNA of 50 familial/early-onset CRC patients, and from formalin-fixed paraffin-embedded (FFPE) normal colon tissues obtained from 632 metastatic CRC patients (details in Suppl. Material and Methods).

Variants with a population minor allele frequency > 0.1% (non-Finnish European gnomAD v4.0.0) were filtered out. Frameshift, stop-gain, canonical splice site and start-loss variants, as well as missense variants with a REVEL pathogenicity score > 0.644 [18], were considered. The frequency of *LTBP4* variant alleles in CRC patients was compared to the allele frequency obtained in gnomAD v4.0.0 control individuals, using the same filtering parameters. Based on the origin of the cases (Spain and the Netherlands), we used as control population the gnomAD v4.0.0 non-Finnish European subset, which includes a total of 590,031 individuals.

### Prediction of HRD from H&E tumor histology slides

To predict HRD from histological slides, two hematoxylin and eosin (H&E)-stained slides from P-8’s tumor were scanned and preprocessed. The slides were first tessellated into 256 µm patches with a resolution of 224 × 224 pixels, followed by feature extraction using a self-supervised learning model (ResNet-50), resulting in 2048-dimensional feature vectors for each patch. For HRD detection, a pretrained deep learning (DL) classifier, based on an attention-weighted multiple instance learning (attMIL) approach, was deployed. Prediction models were trained using a publicly available DL pipeline (https://github.com/KatherLab/marugoto), as previously described [19, 20]. The DL model had been trained on 460 lung adenocarcinoma samples, where HRD was calculated using scarHRD from whole-genome sequencing data [21]. The classifier was then applied to the two processed slides to generate a prediction score for HRD status.

### Tumor multi-gene panel analysis

TruSight Oncology 500 (TSO500) gene panel (Illumina, San Diego, CA), which includes 523 cancer-related genes, was used to sequence DNA extracted from the FFPE material obtained from an H&E-stained slide. DNA extraction was performed with QIAamp DNA FFPE Tissue Kit (Qiagen). DNA libraries were prepared using the hybrid capture-based TSO500 Library Preparation Kit (Illumina). A pooled library from eight DNA samples was loaded on a NextSeq 500/550 High Output Kit v2.5 (300 cycles), and sequencing (paired-end reads; 2 × 101 bp) was performed on an Illumina NextSeq 550 Dx instrument. Raw data were analyzed using the Illumina® TSO500 Local App version 2.2.0.2 in a Docker container. GRCh37/hg19 was used as reference human genome. Results included MMR status, tumor mutational burden, SNVs and small indels, splice-altering variants, gene amplifications, and gene fusions.

#### Mutational signature analysis

The contribution of COSMIC SBS mutational signatures was calculated using FitMS [22], through the Signal web application (https://signal.mutationalsignatures.com/), and with SigProfilerAssignment [23], available on the COSMIC website (https://cancer.sanger.ac.uk/signatures/assignment/). When using Signal, the default SBS signature panel was applied, both with and without selection for tissue-, i.e., colon-, specific signatures (analysis performed in July 2024). Sequencing data from  the TSO500 multi-gene panel and WES data from 42 randomly selected MMR-proficient TCGA CRC samples (COADREAD dataset) were analyzed.

## Results

### Constitutional genome-wide methylation analysis in genetically unexplained familial  and/or early-onset CRC patients

Genome-wide methylation analysis using the Illumina Infinium MethylationEPIC BeadChip was performed in 46 MMR-proficient nonpolyposis CRC patients without germline pathogenic variants in known hereditary cancer genes. The analysis identified several regions—nine of which were located within CpG islands—that were hypermethylated in at least one individual compared to the other 45 patients (Suppl. Table 2). Based on the location of these methylated CpG islands in known hereditary cancer genes, or in genes involved in key colorectal cancer pathways, two of the nine hypermethylated cases were prioritized for further investigation: one with *BRCA1* promoter methylation, and another with methylation of a CpG island in *LTBP4*.

### Constitutional methylation of CpG island 102 in *LTBP4* in an early-onset CRC patient

**Fig. 2:**
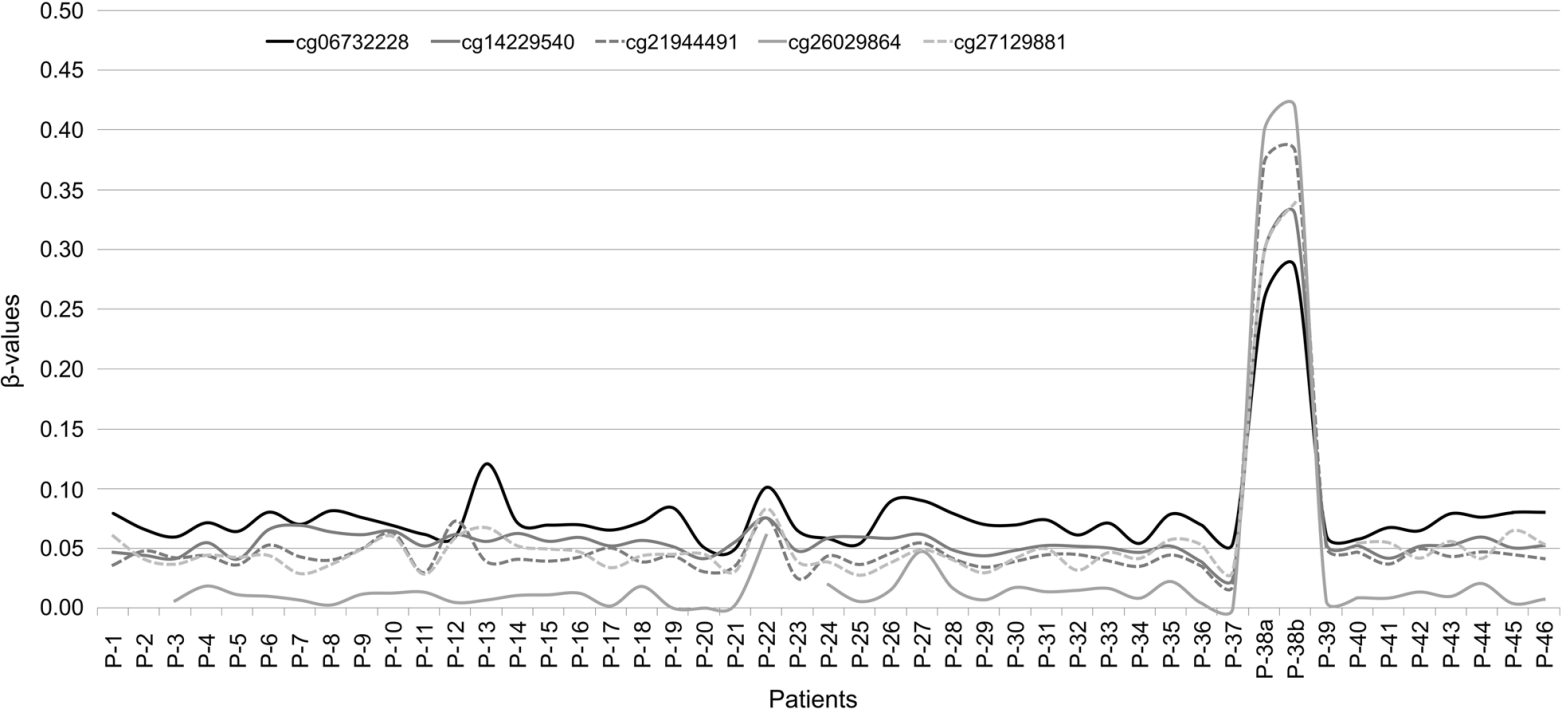
Methylation β-values (Y-axis) of the five CGs that represent CpG island 102 on the Illumina Infinium MethylationEPIC array. The 46 patients analyzed are represented in the X-axis. For patient P-38, two independent samples collected at different time points were included

Two peripheral blood samples collected two years apart from a patient diagnosed with CRC at age 46 (P-38) exhibited an aberrant methylation pattern at CpG island 102, represented by five CpG sites in the Infinium MethylationEPIC array, located within the *LTBP4* gene. The average β-value across these CpGs was ~ 0.34 (range: 0.26–0.42), which suggested constitutional methylation of one allele, i.e., monoallelic methylation (Fig. 2). The observed methylation was confirmed by direct bisulfite sequencing (Suppl. Figure 1A).

**Fig. 3:**
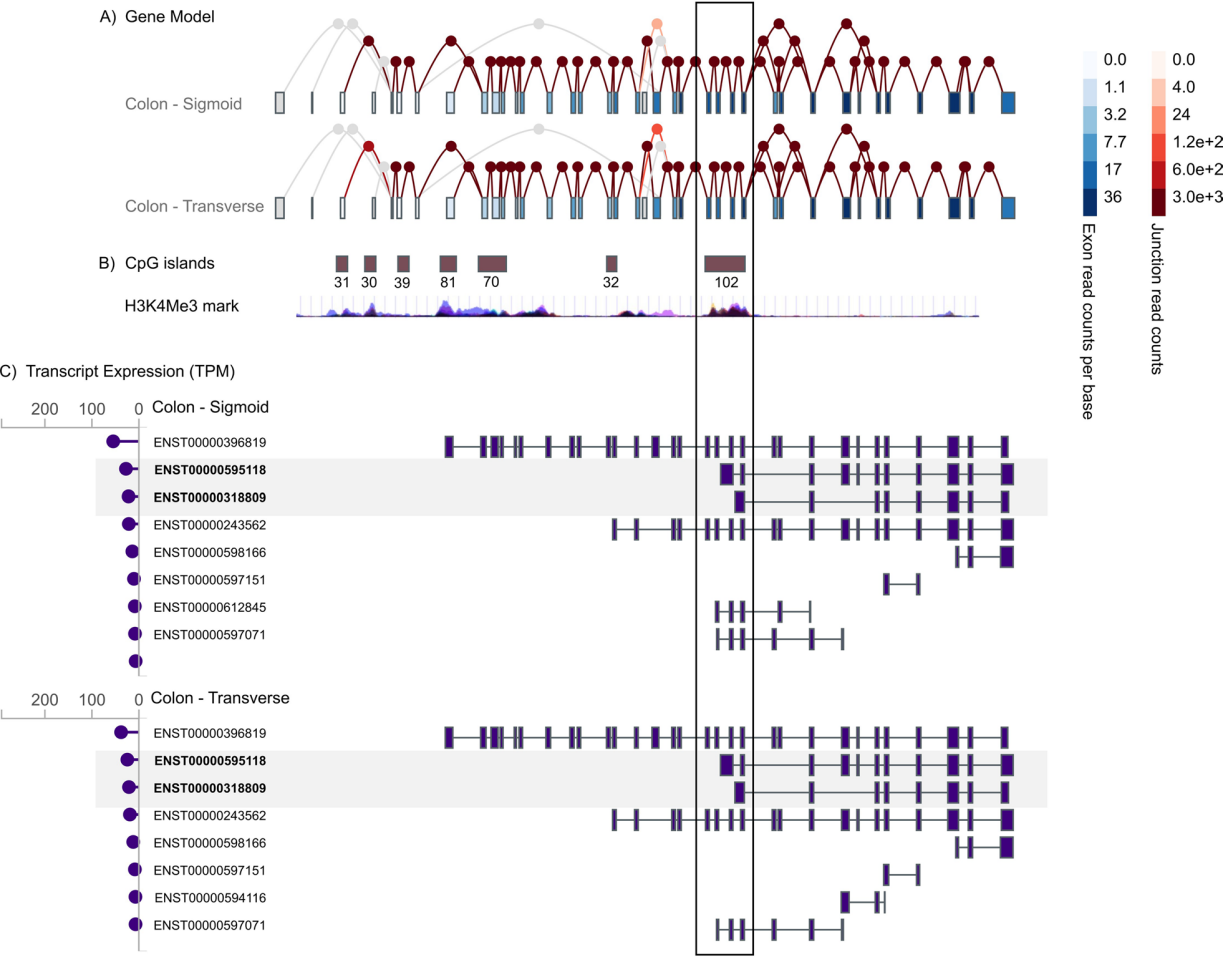
**A** Representation of *LTBP4* exons and alternative splicing for both transverse and sigmoid (left) colon. **B** CpG islands and H3K4Me3 marks in the *LTBP4* genomic region (source: UCSC genome browser). **C** *LTBP4* transcripts represented according to their level of expression (in transcripts per million, TPM) in the transverse and sigmoid (left) colon (source: GTEx portal). Location of CpG island 102 is highlighted with a square

Human *LTBP4* produces several transcripts, including two main isoforms: ENST00000396819 (NM_001042545) and ENST00000308370 (NM_001042544), each regulated by distinct promoters associated with CpG islands 81 and 31, respectively. The presence of multiple CpG islands within the first exons of several *LTBP4* transcripts, together with the presence of H3K4Me3 marks—typically located near active promoters—suggests that other *LTBP4* transcripts are also functional. Specifically, CpG island 102 maps to a predicted promoter region of transcripts ENST00000595118 and ENST00000318809, which are the second and third most highly expressed transcripts in colon normal mucosa, according to GTEx data (Fig. 3). Based on this, constitutional methylation of CpG island 102 is expected to downregulate the expression of these two *LTBP4* transcripts.

The pathology report classified the patient’s tumor as a low-grade adenocarcinoma (pT4a pN2a) located in the sigmoid colon. The tumor arose from a pre-existing villous adenoma, contained more than 50% mucinous component, and showed preserved expression of MLH1, MSH2, MSH6, and PMS2 proteins. No polyps were identified. Unfortunately, no tumor sample was available to perform somatic studies, such as assessment of a potential *LTBP4* second hit and/or expression studies. The proband’s mother, diagnosed with CRC at the age of 69, showed no methylation of *LTBP4* CpG island 102 in blood DNA, as assessed by direct bisulfite sequencing. The proband’s maternal grandmother and her sister were also diagnosed with CRC in their 80s, but no samples were available for analysis (Suppl. Figure 1B). No clinical information or biological material could be obtained from the paternal side of the family, preventing confirmation of a potential de novo origin of the epimutation. WES of blood DNA from the proband and his mother identified no genetic variants classified as pathogenic, likely pathogenic or of uncertain significance in known hereditary genes.

Analysis of the complete *LTBP4* genomic region (WGS data), including 1 Mb upstream the open reading frame (ORF) of the transcripts regulated by CpG island 102, did not identify any genetic alteration or large rearrangement (present in the proband and absent in the unmethylated mother) that could have caused the methylation of one of the *LTBP4* alleles, suggesting a primary epimutation (Suppl. Table 3).

### CpG island 102 somatic methylation correlates with *LTBP4* downregulation in colorectal cancers

Methylation analysis of stage II colon cancer samples (not treated with chemotherapy) and paired normal colon tissues from 90 patients, along with 39 normal colon biopsies from cancer-free individuals—available through the Colonomics browser (www.colonomics.org)—revealed that CpG island 102 is frequently methylated in CRCs but not in the normal colon mucosa of CRC patients or healthy controls. CpG island 102 showed the strongest significant difference in hypermethylation in CRC samples compared to normal colon tissues (Suppl. Figure 2 and Suppl. Table 4). The common occurrence of CpG island 102 somatic methylation in CRC samples was confirmed in 736 CRC samples included in The Cancer Genome Atlas (TCGA; COADRED cohort) (Suppl. Figure 3).

**Fig. 4:**
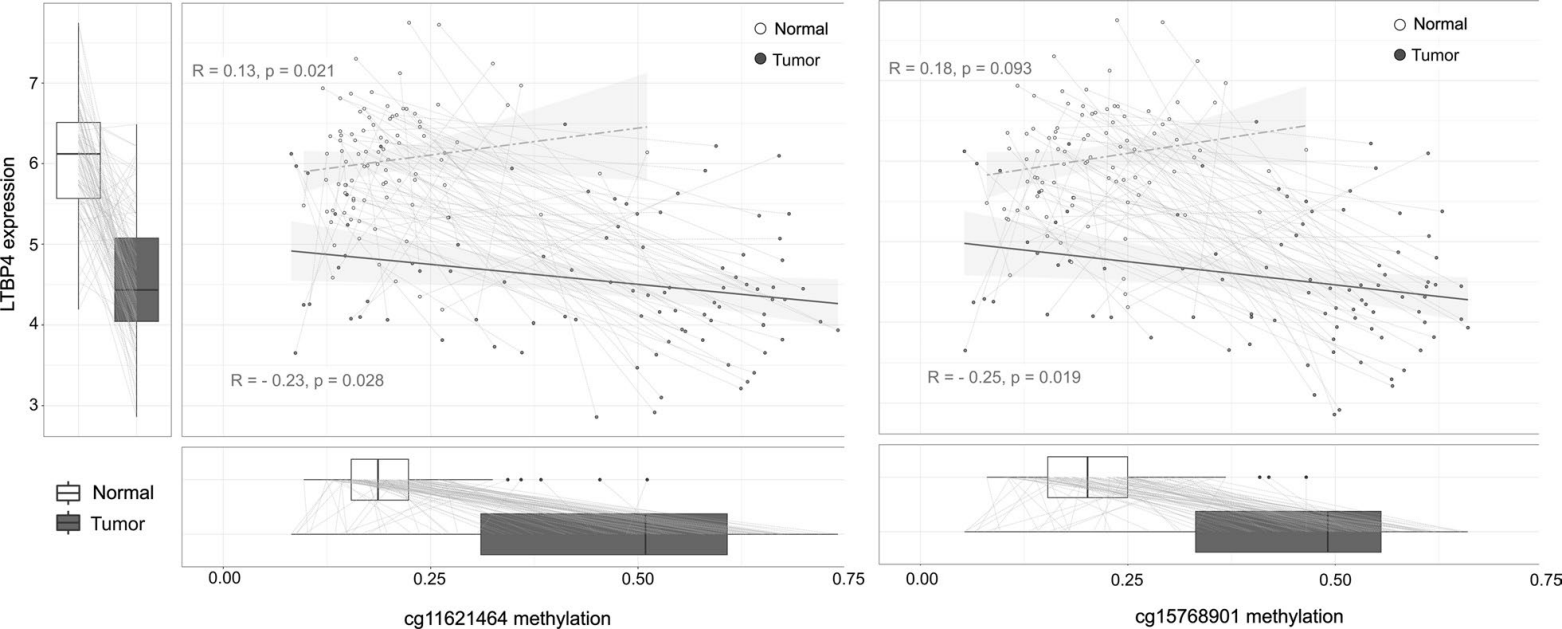
*LTBP4* global expression levels in 90 CRCs and their matched adjacent normal colon mucosa, and correlation with methylation levels at cg11621464 and cg15768901, which are two CpG sites located at the 3' end of CpG island 102

We also observed that *LTBP4* RNA expression was significantly reduced in CRCs relative to their matched normal colon mucosa (Suppl. Figure 4; source: www.colonomics.org). Notably, *LTBP4* global expression reduction in colon cancers, compared to paired adjacent normal colon mucosa, strongly correlated with the methylation of CpG island 102, particularly with the CpG sites located in its 3’end of the island (Fig. 4; Suppl. Figure 4). These findings support a model in which methylation of CpG island 102 contributes to the transcriptional downregulation of *LTBP4* in CRC.

### Constitutional *LTBP4* CpG island 102 methylation and germline *LTBP4 *variants in unsolved familial and/or early-onset colorectal cancer patients

Direct bisulfite sequencing targeting *LTBP4* CpG island 102 was performed on bisulfite-converted blood DNA from 230 familial and/or early-onset CRC patients without pathogenic variants in known hereditary CRC and polyposis genes. No additional cases with constitutional methylation of the CpG island 102 in *LTBP4* were identified, suggesting that this epigenetic alteration is a very infrequent cause of CRC predisposition.

To further explore a potential role of *LTBP4* inactivation in CRC predisposition, we assessed the presence of rare germline *LTBP4* damaging or predicted damaging variants in two cohorts: i) 36 Dutch and 14 Spanish familial and/or early-onset MMR-proficient nonpolyposis CRC patients without  germline pathogenic variants in known hereditary cancer genes; and ii) 632 Dutch patients with metastatic CRC. No damaging or predicted damaging *LTBP4* variants were detected among the 50 familial and/or early-onset CRC cases. In the metastatic CRC cohort, four predicted damaging (REVEL > 0.644) missense variants (c.2162G > A;p.Cys721Tyr, c.3328G > A;p.Glu1110Lys, c.4141G > T;p.Ala1381Ser and c.4802G > A;p.Cys1601Tyr) were identified in four individuals (4/632: 0.64%). However, damaging and predicted damaging *LTBP4* variant alleles were not enriched in CRC patients overall (4/1264; MAF = 0.32%) when compared to their frequency in population controls (5,542/1,118,062; MAF = 0.47%; source: 590,031 non-Finnish European gnomAD v4.0.0.). Notably, no loss-of-function variants (e.g. stop-gain, frameshift, start-loss or canonical splice-site mutations) were identified in any patient cohort, limiting the ability to draw conclusions about the contribution of *LTBP4* germline mutations to CRC predisposition.

### Low-level constitutional methylation of the *BRCA1* promoter in an early-onset CRC patient

**Fig. 5:**
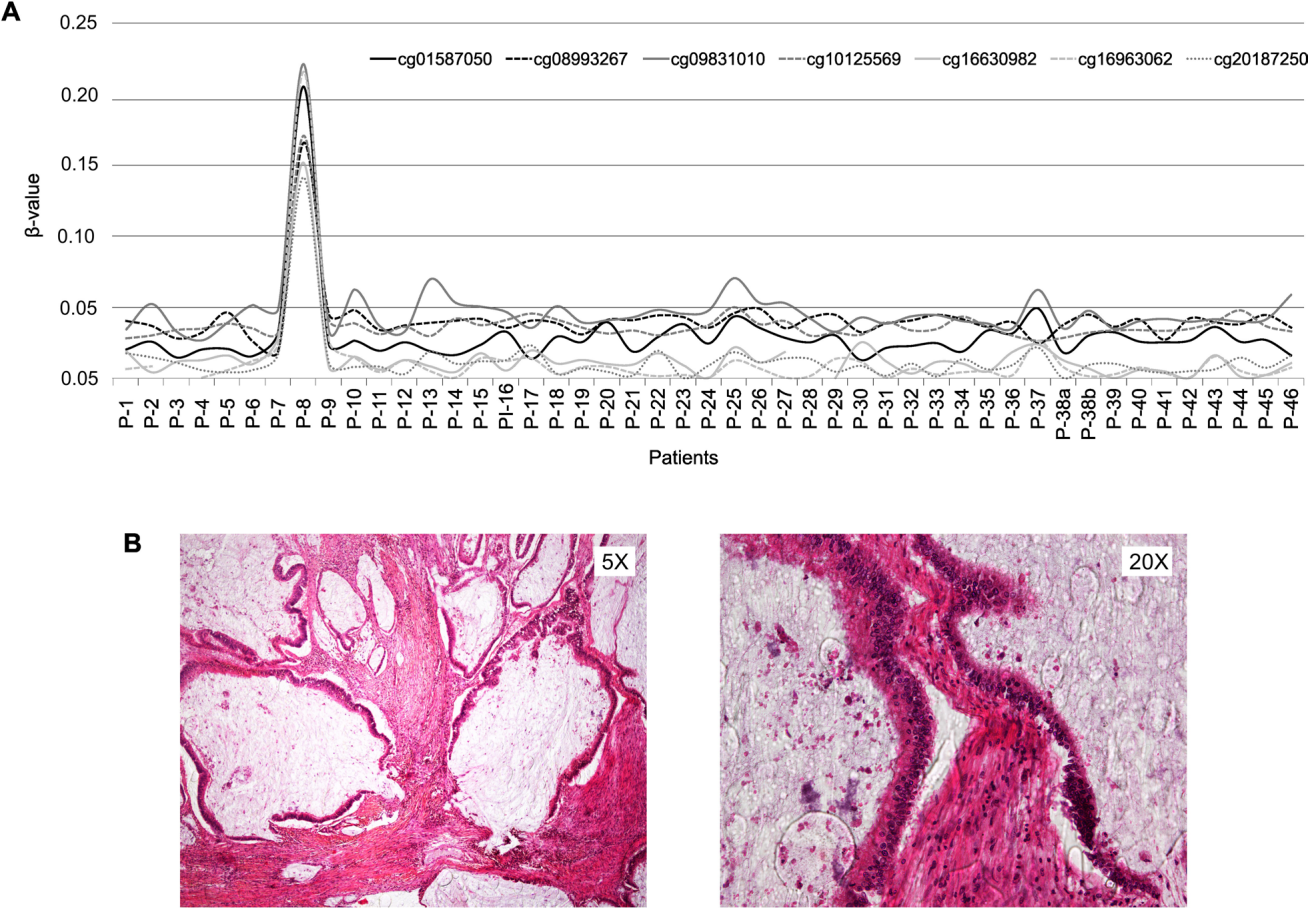
**A** Methylation β-values (Y-axis) of the seven CGs included in the predicted main *BRCA1* promoter. The 46 patients analyzed are represented in the X-axis. **B** Hematoxylin-eosin-stained tissue sections of the colon cancer of P-8

In the genome-wide methylation analysis of the 46 unsolved familial and/or early-onset CRC patients, low-level methylation of the *BRCA1* promoter (average β-value: 0.18; range β-value: 0.14–0.22) was detected in a male individual diagnosed with CRC at 37 years of age and with no reported family history of cancer (P-8) (Fig. 5A). The obtained results were validated by pyrosequencing using the Qiagen PyroMark CpG Assay PM00039907 (methylation level in blood DNA: 13.75%). The concordant results from both platforms are consistent with *BRCA1* promoter methylation mosaicism.

Only two H&E-stained tissue sections were available from the tumor of patient P-8. Histologically, the tumor was identified as a mucinous adenocarcinoma, composed entirely of extracellular mucinous pools surrounded by atypical columnar epithelial cells, consistent with an intestinal origin. No intraepithelial lymphocytosis was observed. In some regions, fibrous desmoplastic stroma was present among dilated mucinous glands (Fig. 5B).

To evaluate the somatic methylation status of the *BRCA1* promoter, methylation levels were assessed in both tumor and blood DNA using pyrosequencing. Similar methylation levels were observed in both samples (12% in tumor, 13.75% in blood;Suppl. Figure 5), suggesting no somatic methylation of the other *BRCA1* allele, or higher methylation levels in colon tissue.

A deep learning classifier trained to detect homologous recombination deficiency (HRD) in lung cancer H&E samples [20] did not detect HRD in this tumor. Due to the rarity of HRD in CRC, a colon-specific model was unavailable. To further explore the possibility of HRD, DNA extracted from one tumor H&E section was sequenced using the Illumina TruSight Oncology 500 (TSO500) multi-gene panel. Analysis of the tumor sequencing data detected neither a second pathogenic variant nor LOH affecting *BRCA1*. After filtering out potential germline variants, a total of 17 somatic variants were identified in the genes covered by the TSO500 panel. This limited number of variants was insufficient to support a robust mutational signature analysis. Despite this limitation, we proceeded to evaluate the tumor’s mutational profile using Signal (FitMS) [22] and SigProfilerAssignment [23]. Neither tool confidently identified HRD-associated mutational signatures. However, Signal suggested, though subthreshold, the presence of HRD-related SBS3 and SBS8 signatures (Suppl. Figure 5).

To assess the presence of HRD-related signatures SBS3 and SBS8 in CRCs, and identify potential *BRCA1*-related etiologies, 42 randomly selected TCGA MMR-proficient CRCs were analyzed, using Signal and the same analytic pipeline used for the patient's tumor data. One case (TCGA-AA-3979) showed SBS3, contributing 32% of the mutational profile, although no promoter methylation, mutation, copy number or structural variants affecting *BRCA1* or other homologous recombination genes were identified (Suppl. Table 5; Suppl. Table 6). Another tumor (TCGA-CI-6624) had a contribution of 28% of SBS8, with two somatic missense variants in *BRCA2* of unknown pathogenic effect, as well as one each in *RAD51AP2* and *BRCC3*.

## Discussion

Our study identified constitutional—likely monoallelic—methylation of a promoter in the *LTBP4* gene (CpG island 102) in a patient diagnosed with CRC at age 46. CpG island 102 potentially regulates two *LTBP4* transcripts expressed in colon epithelium, and the evidence gathered from tumor expression and methylation data suggests that methylation of this island drives global *LTBP4* down-expression in CRC. Through the analysis of the patient’s genomic data, we did not identify a genetic alteration in the *LTBP4* genomic region that could have led to the allele-specific methylation, which suggests that the constitutional CpG island 102 methylation is likely primary in origin.

*LTBP4* encodes an extracellular matrix glycoprotein involved in TGF-β signaling. A dual role has been proposed for LTBP4: as a structural component of the extracellular matrix and as a local regulator of TGF-β tissue deposition and signaling. The TGF-β/BMP pathway plays a relevant role in colorectal carcinogenesis, and particularly in CRC predisposition. Several genes implicated in hereditary colorectal cancer syndromes—such as *SMAD4*, *BMPR1A*, and *GREM1*—are part of this pathway and are associated with hamartomatous and mixed polyposis syndromes [24]. Nevertheless, the patient did not present with polyposis and the CRC was an adenocarcinoma that developed from a pre-existing villous adenoma. On the other hand, CRC risk loci identified by genome-wide association studies (GWAS) include several variants that regulate the expression of TGF-β/BMP signaling components such as *GREM1*, *BMP2*, *BMP4*, *SMAD7*, and *SMAD9* [25, 26].

Supporting the role of *LTBP4* inactivation in CRC predisposition, *Ltbp4* double knockout (KO) mice develop colorectal adenomas and carcinomas, along with severe pulmonary emphysema and cardiomyopathy [27]. These abnormalities are linked to profound defects in the elastic fiber structure and reduced deposition of TGF-β in the extracellular space. As consequence, epithelial cells exhibit decreased levels of phosphorylated Smad2 proteins, overexpress c-myc, and undergo uncontrolled proliferation [27]. In humans, biallelic *LTBP4* pathogenic variants cause an autosomal recessive syndrome (OMIM: 613177) characterized by cutis laxa, early childhood-onset pulmonary emphysema, peripheral pulmonary artery stenosis, and other evidence of a generalized connective tissue disorder such as inguinal hernias and hollow visceral diverticula [28]. No reports of cancers in biallelic carriers have been registered, likely due to the early lethality of the condition (average survival: 2.4 years) and its extreme rarity (25 patients from 20 families described worldwide). All published reports are focused on the clinical description of biallelic carriers, with no mention of health issues in heterozygous relatives. In our study, none of the tested CRC patients carried loss-of-function variants in *LTBP4*. Consequently, we are unable to draw any conclusions regarding the role of monoallelic inactivation of *LTBP4* through mutation in CRC predisposition. Whether CRC predisposition occurs exclusively through constitutional methylation of *LTBP4* CpG island 102 remains to be established.

As noted above, both *LTBP4* biallelic mutant mice and humans exhibit pulmonary emphysema among other phenotypes. In line with this, a pathology report from a pulmonary resection performed on patient P-38—who harbors the constitutional monoallelic methylation of *LTBP4* CpG island 102—dated to the year of the CRC diagnosis, described emphysematous changes and vascular congestion in the lung parenchyma, along with a fibrous area in the pleura. These findings might be related to the *LTBP4* deficiency.

*LTBP4* downregulation has been reported in gastrointestinal cancers [29] and other tumor types [30–32]. In this study we confirmed the *LTBP4* down-expression in CRC by analysing gene expression data from colon tumors and matched normal colon mucosa of 98 untreated stage II colon cancer patients (Fig. 4; source: https://www.colonomics.org/). Furthermore, we demonstrated that *LTBP4* downregulation in CRC strongly correlates with methylation of CpG island 102, particularly at CpG sites located at the 3’ end of the island. A prior study showed that treatment of esophageal adenocarcinoma and squamous cell carcinoma cell lines with demethylating agents resulted in *LTBP4* upregulation and decreased cancer cell migration [29].

In the analyzed family, although three additional CRC cases were diagnosed on the maternal side (mother, grandmother, and grandaunt, at ages 69, 88, and 82, respectively), constitutional methylation of  *LTBP4* CpG island 102 was observed exclusively in the proband. Although these diagnoses occurred at advanced ages, the aggregation of cancers suggests the presence of genetic CRC (low-)risk factors or shared lifestyle or environmental exposures that increased the risk of CRC in these relatives. Irrespective of the underlying cause of this familial clustering, the *LTBP4* CpG island 102 methylation in the proband may have elevated the baseline risk, precipitating CRC onset at a considerably younger age (46 years).

The underlying cause of the apparent de novo methylation event at *LTBP4* CpG island 102 in patient P-38 remains unresolved. Although unlikely, given that methylation of CpG island 102 was the only constitutional epimutation identified in P-38, we investigated whether the patient harbored germline variants in genes involved in methylome maintenance  which might underlie the de novo hypermethylation event. WES and WGS data were screened for variants in 32 genes involved in DNA methylation pathways/processes, as listed in the Reactome database. We considered variants with a minor allele frequency (MAF) < 1% in gnomAD v4.1.0. Only one variant was identified: a non-coding intronic variant in *DNMT3B* (NM_006892.4: c.1297 + 5C > T; p.?), which is not predicted to affect splicing by SpliceAI. This variant has a gnomAD v4.1.0 MAF of 0.0016%, has been submitted twice to ClinVar (classified as a variant of uncertain significance and likely benign), and has not been reported in individuals with *DNMT3B*-related disorders. Given its intronic location and absence of predicted functional impact, this variant is unlikely to be pathogenic.

In addition to the *LTBP4* constitutional monoallelic methylation, our analysis also identified mosaic constitutional methylation of the *BRCA1* promoter in a male patient diagnosed with CRC at age 37 and with no family history of cancer. Beyond female breast and ovarian cancers, germline pathogenic variants in *BRCA1* have also been associated with elevated risks of male breast cancer, pancreatic, and gastric cancers, and associations with colorectal and gallbladder cancers have been suggested [2, 33]. Constitutional mosaic epimutations in *BRCA1* have been linked to increased risk of breast and/or ovarian cancer [9, 34, 35]. In P-8, the methylated region overlaps the 5’UTR region which,  when methylated, is associated with an increased predisposition of breast and ovarian cancer. Prior studies have demonstrated that methylation in this region results in monoallelic silencing of the affected allele (*in cis*) [36, 37]. Whether *BRCA1* deficiency is the driver of the early-onset CRC in this patient remains unclear. Tumor-based analyses yielded inconclusive results: although mutational signature analysis suggested a certain level of HRD, no somatic second hit in *BRCA1*—via LOH, mutation or promoter methylation—was detected.

Several studies have reported a marginal increase in CRC risk among heterozygous carriers of *BRCA1* pathogenic variants. A recent study evaluating second primary cancer risks in over 25,000 *BRCA1* pathogenic variant carriers diagnosed with breast cancer found a significantly increased risk of CRC compared to population incidence rates [38]. Other large studies, including a meta-analysis, have also suggested a small increase in CRC risk associated with *BRCA1*, while, in general, no consistent association has been observed for *BRCA2* [2, 33, 39–41]. Beyond the contribution of *BRCA1* deficiency to genomic instability due to impaired homologous recombination, it has been proposed that BRCA1 may influence CRC risk through its role in oxidative DNA damage repair, particularly through the activation of key base excision repair components [42, 43].

## Conclusions

Our findings suggest that constitutional (monoallelic) methylation of *LTBP4* CpG island 102 is associated with an increased risk of early-onset CRC. This CpG island regulates the expression of two *LTBP4* transcripts that are particularly relevant in the colonic epithelium. Although rare as a constitutional event, methylation of *LTBP4* CpG island 102 is commonly observed as a somatic alteration in CRC, where it contributes to global *LTBP4* downregulation. Supporting a causal role in CRC predisposition, *LTBP4* KO mice develop colorectal tumors. In addition to early-onset CRC, pulmonary emphysema—and possibly other phenotypes associated with *LTBP4* deficiency—may also occur in individuals with monoallelic methylation of *LTBP4* CpG island 102, potentially offering clues to the underlying constitutional epigenetic defect.

We have also identified mosaic constitutional methylation of the *BRCA1* promoter in a patient diagnosed with early-onset CRC. Whether *BRCA1* inactivation is the carcinogenic driver of the colorectal tumor in that patient remains unresolved, and further studies are needed to determine its potential role as a CRC predisposing factor.

## Supplementary Information

Additional file1 (PDF 1230 KB)

## Data Availability

Data supporting the reported results, analytical methods and study materials may be found in the article or supplementary material. Colonomics (www.colonomics.org) data used for this analysis are available at the CORA research data repository (doi:10.34@810/data169). Whole-exome sequencing (WES) data from Spanish patients is stored at the European Genome-phenome Archive (EGAD00001009767). Restrictions are in place for access to the remaining WES and raw whole-genome sequencing (WGS) data because of lack of consent for data sharing. Additional data or information will be made available upon request.

## Abbreviations

CGI: CpG island
CRC: Colorectal cancer
DL: Deep learning
FFPE: Formalin-fixed paraffin-embedded
gnomAD: Genome aggregation database
GWAS: Genome-wide association studies
H&E: Hematoxylin and eosin
HRD: Homologous recombination deficiency
MMR: DNA mismatch repair
N: Normal (non-cancer) tissue
ORF: Open reading frame
PCA: Principal component analysis
SNV: Single nucleotide variant
T: Tumor (cancer) tissue
TSO500: TruSight Oncology 500 multi-gene panel
WES: Whole-exome sequencing
WGS: Whole-genome sequencing
