# Supplementary material for: Constitutional epimutations in LTBP4, a component of the TGF-β signaling, and in BRCA1, as potential drivers of early-onset colorectal cancer
Source: Clin Epigenetics. 2025 Nov 5;17:183. doi: 10.1186/s13148-025-01924-x (PMC12587707; doi:10.1186/s13148-025-01924-x)
Supplement: Supplementary file 1 — Additional file1 (PDF 1230 KB) [file 13148_2025_1924_MOESM1_ESM.pdf]

SUPPLEMENTARY MATERIAL AND METHODS

Whole-exome and whole-genome sequencing of colorectal cancer patients

Dutch patients

A total of 692 individuals diagnosed with colorectal cancer were included in this study. Sixty of them were genetically unexplained, pMMR, familial, and/or early-onset CRC patients that belonged to 36 families and that were subjected to whole-exome sequencing (blood DNA). The remaining 632 individuals had metastasized CRCs with available germline and tumor whole-genome sequencing (WGS) data, accessible upon request through the Hartwig Medical Foundation database (reference number HMF-DR-288; https://www.hartwigmedicalfoundation.nl/). Anonymized tissue samples were handled following the medical ethical guidelines outlined in the Code of Conduct for responsible use of human tissue in the context of health research (Federation of Dutch Medical Scientific Societies). The study was approved by the Medical Ethical Committee of the Leiden University Medical Center (The Netherlands) (Protocol P01.019).

Exome library preparation and sequencing were carried out at GenomeScan (Leiden, The Netherlands), as previously described.[1] SureSelect Human All Exon V5 (Agilent Technologies) was used to perform the exome enrichment, following the manufacturer´s instructions, and the obtained libraries were sequenced on a HiSeq2500 sequencer (Illumina, Inc).

For a detailed overview of the whole-genome sequencing methodology for the mCRCs, please refer to the documentation provided by the Hartwig Medical Foundation database (https://hartwigmedical.github.io/documentation/data-access-request-methods.html#dna-sequencing-workflow). In short, DNA isolation from biopsy and blood was performed using an automated QiaSymphony setup, adhering to Qiagen's protocols. The TruSeq Nano LT library preparation (Illumina) took place on an automated liquid handling platform (Beckman Coulter). Barcoded libraries were subsequently sequenced on HiSeq X (V2.5 reagents) and Novaseq 6000 S4 Reagent Kit (Illumina). BCL output underwent conversion using the bcl2fastq tool (Illumina). Reads were aligned to the reference genome GRCh37 using BWA-mem v0.7, with duplicate reads marked for filtering and INDELs realigned through GATK v3. GATK HaplotypeCaller v3 was utilized for calling germline variants in the reference sample, while SAGE v2 was employed to call somatic SNVs and small INDELs.

Spanish patients

Exome library preparation and sequencing were carried out at CNAG (Barcelona, Spain), as previously described.[2] SureSelect Human All Exon V5 (Agilent Technologies) was used to perform exome enrichment following the manufacturer´s instructions. In brief, 2.5-3.0 ug of genomic DNA was sheared on Covaris instrument LE220 and size selected with AMPure XP beads (Agencourt, Beckman Coulter). The fragmented DNA was end-repaired, adenylated, and ligated to Agilent sequencing adaptors. The DNA with adaptor-modified ends was pre-capture amplified with 6 PCR cycles (Herculase II fusion DNA polymerase, Agilent Technologies). The PCR product was quality controlled on the Agilent 2100 Bioanalyzer 7500 chip (Agilent Technologies). The hybridization mix was washed, and the eluate was post-capture PCR amplified (12 cycles)

to add the index tags using SureSelectXT Indexes for Illumina. The final library size and the concentration was determined on Agilent 2100 Bioanalyzer 7500 chip.

The captured libraries were sequenced on a HiSeq2500 sequencer (Illumina, Inc) in paired-end mode with a read length of 2x101bp using TruSeq SBS v4 chemistry. Each sample was sequenced in a fraction of a sequencing flow cell lane, following the manufacturer's protocol. Image analysis, base calling and quality scoring of the run were processed using the manufacturer's software Real Time Analysis (RTA 1.18.66.3) and followed by generation of FASTQ sequence files.

FASTQ files from exome sequencing analyses were aligned to the human reference genome (GRCh37) using BWA-MEM[3] (https://arxiv.org/abs/1303.3997). Bamsort was used to convert files into bam. Afterwards, duplicate sequences were marked using bammerge and bammarkduplicates2 tools in cases where there were more than one pair of fastq files. IGV was used for bams visualization. Variant calling was done by running HaplotypeCaller (GATK4) individually for each sample. After results normalization using GATK4, two files containing Indels and SNVs were obtained. Finally, to ensure data quality, the following quality filters were applied: DP (Read Depth) < 8; FS (Fisher Strand) > 25.0; QD (Quality by Depth) < 6.0 and MQ (RMS Mapping Quality) < 50.0.

SUPPLEMENTARY TABLES

Supplementary Table 1. Characteristics of the 46 MMR-proficient non-polyposis familial and/or early-onset unrelated colorectal (CRC) patients included in the study.

	n	Mean age at cancer diagnosis	Median age at cancer diagnosis	Age range
**Proband affected with CRC**				
Amsterdam [I / II]	22 [21/1]	43.05	42	32-59
Bethesda	24	37.54	37.50	28-47
**TOTAL**	46	40.17	40	28-59
**Proband affected with other tumor types**				
Amsterdam [I / II]	1 [1/0] [a]	47	47	47
Bethesda	1 [b]	28	28	28

a. Proband diagnosed with cervix cancer at age 47
b. Proband diagnosed with a second CRC at age 30

Supplementary Table 2. List of differentially methylated CpGs and CpG islands (CGI) identified in the 46 patients using the Illumina Infinium MethylationEPIC array. The table includes the β-values of the methylated CpGs in the corresponding patients and the mean β-value of the CpG in the other 45 patients.

Patient	Gene	CpG	CpG island	β-value	
				Patient	Other 45 patients (mean)
P-6	*WIBG*	cg00060880	CGI 26	0.3696	0.0688
		cg01157152		0.4572	0.0215
		cg14304690		0.4997	0.0344
		cg01791737		0.2665	0.0113
		cg22515710		0.5541	0.0353
		cg11369906		0.4393	0.0539
		cg12743743		0.4313	0.0495
		cg13722634		0.4662	0.0165
		cg14032596		0.3817	0.0476
		cg08724866		0.4759	0.0290
		cg17169797		0.0859	0.0061
		cg01694696		0.4490	0.0312
		cg25822535		0.3634	0.0382
P-8	*BRCA1*	cg09831010	Upstream CGI 24	0.2214	0.0439
		cg01587050		0.2052	0.0256
		cg10125569		0.1707	0.0354
		cg16630982		0.1518	0.0108
		cg16963062		0.2157	0.0071
		cg20187250		0.1416	0.0095
		cg08993267		0.1659	0.0380
P-10	*RP13-137A17.5*	cg01263624	No	0.4269	0.0950
		cg17693826		0.4176	0.0205
		cg23987897		0.4876	0.0726
P-16	*C10orf4*	cg00051979	No	0.4163	0.0468
		cg01154537		0.5055	0.0461
		cg04551154		0.3114	0.0376
		cg06152496		0.0740	0.0041
		cg07550278		0.1354	0.0056
		cg07902884		0.3254	0.0283
		cg09068993		0.5255	0.0280
		cg10830758		0.2837	0.0491
		cg16661579		0.1801	0.0064
		cg17233127		0.1231	0.0058
P-17	*SEC23IP*	cg04465599	CGI 48	0.3430	0.0035
		cg04536721		0.3767	0.0658
		cg20448716		0.3912	0.0051
		cg04622176		0.3970	0.0093
		cg06603682		0.2359	0.0072
		cg18363267		0.5173	0.0284
		cg21201659		0.2855	0.0089
		cg25418309		0.3574	0.0395
		cg25377358	No	0.3546	0.0609
		cg05793155		0.3412	0.0306
		cg05909886		0.4464	0.0265
		cg11136434		0.3553	0.0348
	FLJ26850	cg16349876	No	0.2483	0.0623
		cg18756179		0.3108	0.0488
		cg22531183		0.4884	0.0680
P-18	*RP11-1398P2.1*	cg00935307	No	0.1875	0.0254
		cg08604533		0.1614	0.0118
		cg10675865		0.1627	0.0353
		cg23502023		0.1570	0.0131
		cg27636047		0.1721	0.0362
P-19	*ZNF581*	cg07938869	CGI 39	0.3876	0.1333
		cg19861460		0.2435	0.1129
		cg26770479		0.3502	0.1920
P-20	*C2orf70*	cg04917511	CGI 58	0.2663	0.0122
		cg10150686		0.1255	0.0140
		cg11374871		0.2609	0.0531
		cg22734086		0.2288	0.0328
P-25	*MEGF10*	cg05648672	Upstream CGI 63	0.3232	0.0178
		cg10397875		0.3459	0.0334
		cg23932859		0.5219	0.1380
		cg04280969	CGI 63	0.3141	0.0378

Patient	Gene	CpG	CpG island	β-value	
				Patient	Other 45 patients (mean)
P-26	*RNF5P1*	cg06570818	No	0.2805	0.0538
		cg07482220		0.4538	0.0927
		cg10023837		0.2989	0.0180
		cg17455891		0.1454	0.0314
		cg18928683		0.1382	0.0404
		cg27370696		0.2511	0.0374
	AGPAT1	cg01052103	No	0.1864	0.0228
		cg01074928		0.1744	0.0436
		cg08450897		0.1582	0.0312
		cg09301199		0.1986	0.0492
		cg13763617		0.1130	0.0071
		cg15124201		0.2531	0.0404
		cg26340737		0.3252	0.0202
P-27	*ZMAT2*	cg07760720	No	0.2267	0.0320
		cg07889058		0.1731	0.0476
		cg17778434		0.1662	0.0121
		cg20948472		0.1707	0.0283
P-30	*PTPRN2*	cg04064735	No	0.0849	0.8204
		cg11835544		0.1516	0.7482
		cg17737875		0.3923	0.8784
P-33	*LRPAP1*	cg16565635	No	0.0070	0.1264
		cg21752583		0.7797	0.2808
		cg23528723		0.0411	0.3356
P-34	*PHACTR1*	cg00460589	No	0.4628	0.0537
		cg06879394		0.4836	0.0163
		cg07912922		0.4807	0.0521
		cg20827128		0.4241	0.0290
		cg21538684		0.4211	0.0829
P-38[a]	*LTBP4*	cg06732228	CGI 102	0.28457	0.25786
		cg14229540		0.32901	0.29749
		cg21944491		0.38184	0.37188
		cg26029864		0.41859	0.39832
		cg27129881		0.3389	0.29653
P-40	*XXbac-BPG181B23*	cg01290934	No	0.1841	0.0457
		cg18086664		0.1917	0.0452
		cg18892128		0.3345	0.0144
		cg19258508		0.2307	0.0106
		cg23354933		0.1913	0.0233
P-41	*HIST1H4L*	cg06323023	No	0.1202	0.0269
		cg20899581		0.1457	0.0166
		cg25845597		0.1285	0.0305
P-42	*PRRC1*	cg09714181	CGI 64	0.4757	0.0129
		cg18233595		0.2279	0.0204
		cg15851800		0.3429	0.0536
		cg04241501		0.3084	0.0529
		cg22009596	No	0.2908	0.0382
		cg25998860		0.2810	0.0583
		cg05443326		0.3008	0.0411
		cg13590816		0.3243	0.0290
		cg15016296		0.4316	0.0240
P-43	*ATP5E*	cg01503881	CGI 88	0.2217	0.0141
		cg01789728		0.3010	0.0620
		cg11595749		0.1926	0.0508
		cg11919138		0.2306	0.0166
		cg16152482		0.3087	0.0390
		cg16347018		0.1462	0.0196
		cg23831021		0.1907	0.0281
		cg24457521		0.2075	0.0182
		cg26831148		0.2939	0.0572
P-44	*MCCC1*	cg03344955	No	0.2855	0.0528
		cg04909259		0.6945	0.9736
		cg07464924		0.3699	0.0264
		cg22211233		0.2560	0.0564
		cg23476885		0.7049	0.9757
		cg25441771		0.2744	0.0249

a. β-values for P-38 correspond to the mean value obtained from two different blood samples from the patient.

Supplementary Table 3. Rare (MAF<0.1%) variants identified 1Mb upstream (hg38 chr19:39613126-40613126) of *LTBP4* CpG island 102 in patient P-38. Variants identified in the proband and absent in the mother are included. Chromosome position refers to GRCh38 - hg38 genome reference.

Chr	Position	Ref	Alt	Location	Gene	Biotype	HGVSc	Variant	MAF [a]
19	39672225	C	T	Regulatory region	-	enhancer	-	rs569685265	0.054 %
19	39854888	C	T	Intergenic	-	-	-	rs1228648495	0.003 %
19	40248777	T	G	Intron	*AKT2*	protein_coding	ENST00000392038.7: c.288-6090A>C	rs1319524121	0.022 %
19	40604860	C	G	Intron	*LTBP4*	protein_coding	ENST00000396819.8: c.251-175C>G	rs1409761364	0.001 %

a. Source: gnomAD v4.1.0. non-Finnish Europeans

Abbreviations: Alt, alternative allele; Chr, chromosome; HGVSc, Human Genome Variation Society coding sequence name; MAF, minor allele frequency; Ref, reference allele

Supplementary Table 4. Details and statistics (Bonferroni adjusted p-values) of methylation levels between normal and tumor tissue of CpGs included at *LTBP4* CpG island 102 and CpG island 81 of Suppl. Figure 2. Source: www.colonomics.org.

	CpG identification Sample	n	β-value mean	Standard deviation	Bonferroni adjusted p-value
CpG island 102	cg06732228				
	NoCancer	37	0.42	0.05	1.88e-12
	Tumor	96	0.56	0.13	
	cg03309253				
	NoCancer	37	0.15	0.07	< 2e-16
	Tumor	96	0.45	0.24	
	cg27645259				
	NoCancer	37	0.14	0.03	< 2e-16
	Tumor	96	0.41	0.22	
	cg11621464				
	NoCancer	37	0.15	0.07	< 2e-16
	Tumor	96	0.44	0.19	
	cg15768901				
	NoCancer	37	0.14	0.08	< 2e-16
	Tumor	96	0.42	0.18	
	cg26441372				
	NoCancer	37	0.16	0.07	1.74e-08
	Tumor	96	0.28	0.14	
CpG island 81	cg10253929				
	NoCancer	37	0.08	0.01	0.00015
	Tumor	96	0.15	0.13	
	cg22046408				
	NoCancer	37	0.18	0.03	1.63e-06
	Tumor	96	0.32	0.19	
	cg18878134				
	NoCancer	37	0.06	0.01	1
	Tumor	96	0.06	0.02	
	cg12486308				
	NoCancer	37	0.11	0.02	1
	Tumor	96	0.11	0.04	

Supplementary Table 5. Mutational signature analysis in 42 randomly selected MMR-proficient CRC samples from TCGA.

TCGA COADREAD	TMB (mut/Mb)[a]	MMR status[b]	Cosine similarity[c]	Somatic mutations[b,c]		Cosmic SBS mutational signatures[c]			HRD-related mutations[b] (classification according to CGI[d])
				Total	Unassigned to SBS	Signature	Etiology	Contribution	
TCGA-A6-5667	1.9	MSS	0.744	49	7 (14%)	SBS1	Age	86%	
TCGA-AA-3812	3.0	MSS	0.883	94	12 (13%)	SBS1	Age	76%	
TCGA-A6-6142	2.0	MSS	0.856	70	7 (10%)	SBS1	Age	90%	
TCGA-A6-6137	3.5	MSS	0.934	106	1 (1%)	SBS1	Age	99%	
TCGA-AA-3818	3.9	MSS	0.853	120	10 (9%)	SBS1	Age	69%	
						SBS18	BER deficiency	23%	
TCGA-AA-3841	2.1	MSS	0.907	58	3 (6%)	SBS1	Age	94%	
TCGA-AA-3971	2.3	MSS	0.789	70	6 (9%)	SBS1	Age	74%	
						SBS13	APOBEC	17%	
TCGA-AA-3979	3.9	MSS	0.869	114	13 (12%)	SBS1	Age	57%	*FBXW7*: R658* (driver); *TP53*: R248W (driver)
						**SBS3**	**HRD**	**32%**	
TCGA-AA-A004	2.0	MSS	0.871	60	15 (25%)	SBS1	Age	75%	
TCGA-AA-A024	2.9	MSS	0.894	72	15 (21%)	SBS1	Age	79%	
TCGA-AA-A02F	1.7	MSS	0.730	44	15 (35%)	SBS1	Age	65%	
TCGA-AA-A02H	2.2	MSS	0.702	66	21 (32%)	SBS1	Age	68%	
TCGA-D5-6898	2.2	MSS	0.836	63	14 (22%)	SBS1	Age	63%	
TCGA-DM-A0X9	5.5	MSS	0.910	150	22 (15%)	SBS1	Age	85%	
TCGA-F4-6809	3.5	MSS	0.847	135	69 (51%)	SBS1	Age	49%	
TCGA-G4-6315	4.2	MSS	0.940	117	15 (13%)	SBS1	Age	87%	
TCGA-G4-6323	2.9	MSS	0.846	86	8 (10%)	SBS1	Age	90%	
TCGA-5M-AATA	2.2	MSS	0.858	65	1 (1%)	SBS1	Age	99%	
TCGA-A6-5664	1.9	MSS	0.895	58	15 (9%)	SBS1	Age	85%	
TCGA-AA-3519	1.2	MSS	0.796	20	0 (0%)	SBS1	Age	100%	
TCGA-AA-3530	2.2	MSS	0.882	82	7 (9%)	SBS1	Age	91%	
TCGA-AA-3560	1.3	MSS	0.481	18	8 (46%)	SBS1	Age	54%	
TCGA-AA-3688	1.6	MSS	0.842	61	10 (17%)	SBS1	Age	83%	
TCGA-AH-6544	4.2	MSS	0.845	127	47 (37%)	SBS1	Age	63%	
TCGA-CI-6624	7.7	MSS	0.833	232	90 (39%)	SBS1	Age	33%	*BRCA2: D1096N & D1096E; BRCC3: L312H;*
						**SBS8**	HRD and TSB	28%	*RAD51AP2: K92N (all passengers)*
TCGA-CL-4957	2.1	MSS	0.831	64	4 (6%)	SBS1	Age	94%	
TCGA-CM-6675	2.3	MSS	0.771	69	12 (17%)	SBS1	Age	83%	
TCGA-CM-6678	3.4	MSS	0.939	102	1 (1%)	SBS1	Age	99%	
TCGA-D5-6529	2.9	MSS	0.852	88	3 (3%)	SBS1	Age	97%	
TCGA-D5-6535	5.0	MSS	0.876	149	20 (13%)	SBS1	Age	56%	
						SBS18	BER deficiency	30%	
TCGA-D5-6537	3.2	MSS	0.942	95	10 (10 %)	SBS96	*MBD4* deficiency	56%	
						SBS18	BER deficiency	33%	
TCGA-DC-6158	2.7	MSS	0.823	81	21 (26 %)	SBS1	Age	56%	
						SBS2	APOBEC	19%	
TCGA-DM-A28G	3.4	MSS	0.878	101	13 (13 %)	SBS1	Age	87%	
TCGA-G4-6627	3.1	MSS	0.926	93	8 (9 %)	SBS1	Age	91%	
TCGA-QG-A5YW	1.9	MSS	0.901	122	23 (19 %)	SBS1	Age	81%	
TCGA-QG-A5Z1	1.9	MSS	0.908	87	3 (3 %)	SBS1	Age	97%	
TCGA-D5-6924	4.6	MSS	0.944	135	34 (25 %)	SBS1	Age	75%	
TCGA-AA-3685	1.9	MSS	0.919	76	14 (18 %)	SBS1	Age	82%	
TCGA-CA-6718	104.0	MSS	0.957	3104	179 (6 %)	SBS10a	*POLE* deficiency	94%	
TCGA-AA-3869	1.8	MSS	0.885	54	0 (0 %)	SBS1	Age	100%	
TCGA-DM-A28K	3.1	MSS	0.934	91	5 (5 %)	SBS1	Age	95%	
TCGA-AF-2687	3.1	MSS	0.900	94	3 (3 %)	SBS1	Age	97%	

[a] Result obtained using MuSiCa[4]
[b] Obtained from TCGA (https://portal.gdc.cancer.gov/)
[c] Mutational signature analysis using FitMS through the Signal web application (https://signal.mutationalsignatures.com/)[5]
[d] https://www.cancergenomeinterpreter.org/analysis
Abbreviations: BER, base-excision repair; CGI, Cancer Genome Interpreter; HRD, Homologous recombination deficiency; MMR, DNA mismatch repair; MMS, MMR stable; TMB, tumor mutational burden; TSB, transcription-strand bias.

Supplementary Table 6. DNA methylation β-values (Illumina HumanMethylation450 BeadChip) obtained from TCGA portal for the two MMR-proficient CRC with contributions of SBS3 or SBS8 (**Suppl. Table 5**). The analysis was restricted to the *BRCA1* promoter region differentially methylated on P-8, which is the same promoter region that was constitutionally methylated in breast and ovarian cancer cases.[6,7]

CpG	Genomic location (hg38)	TCGA-AA-3979 (β-values)	TCGA-CI-6624 (β-values)
cg08993267	chr17:43125305-43125305	NA	NA
cg24806953	chr17:43125347-43125347	NA	0.0149760646449226
cg20187250	chr17:43125364-43125364	NA	0.0186393945348509
cg15419295	chr17:43125372-43125372	NA	0.0221898542503872
cg16963062	chr17:43125375-43125375	NA	0.0181442295798022
cg16630982	chr17:43125377-43125377	NA	0.0290300248837319
cg21253966	chr17:43125409-43125409	NA	0.0147501945902968
cg04110421	chr17:43125411-43125411	NA	0.035143815296648
cg04658354	chr17:43125427-43125427	0.0236317833774686	0.0291868336143119
cg17301289	chr17:43125445-43125445	NA	0.0408348860989712
cg09441966	chr17:43125470-43125470	NA	0.0345946074960349
cg26891576	chr17:43125524-43125524	NA	0.0632936378302039
cg20760063	chr17:43125563-43125563	NA	NA
cg10893007	chr17:43125677-43125677	0.021231689415829	0.0394371629992124
cg11126247	chr17:43125691-43125691	NA	NA
cg12182452	chr17:43125714-43125714	NA	0.0550303632429261
cg09831010	chr17:43125830-43125830	NA	0.0425882667069609

SUPPLEMENTARY FIGURES

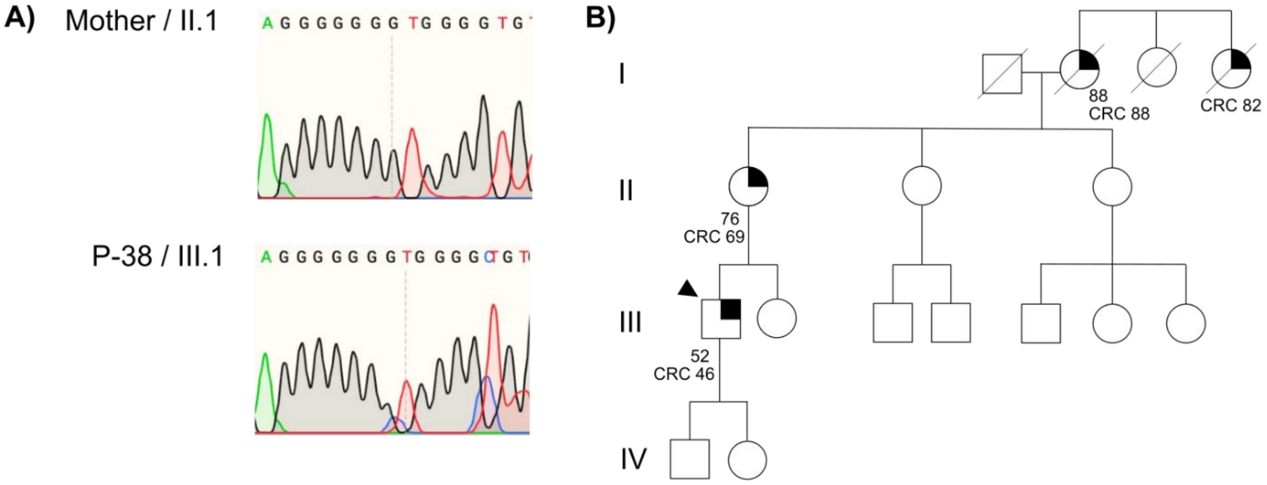

Supplementary Figure 1. Pedigree of the proband (III1) of *LTBP4* monoallelic methylation. Quarter black symbol denotes CRC. Black arrowhead indicates the proband. Whenever available. age at last follow-up or at death is indicated and. below the symbol. the cancer type and age at diagnosis.

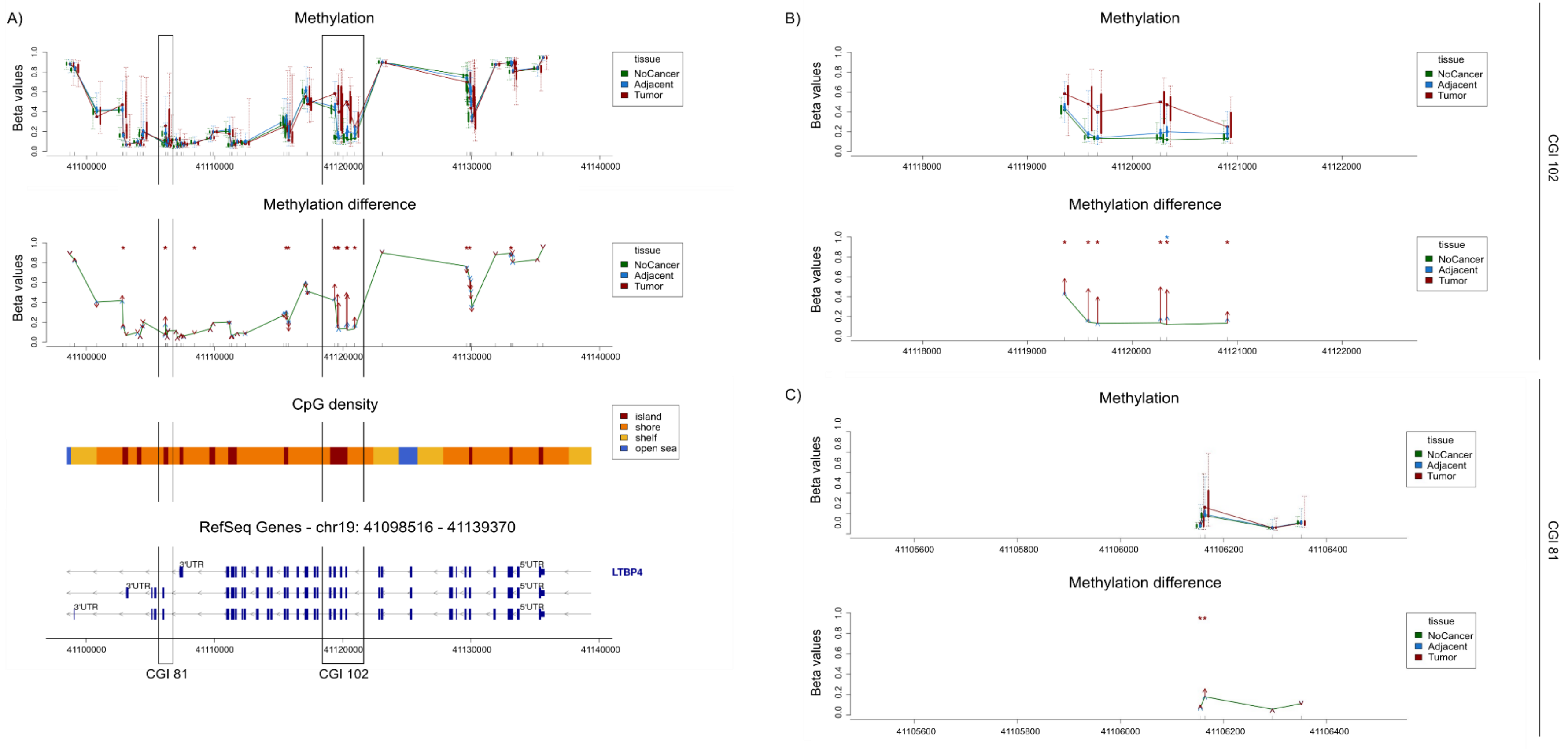

Supplementary Figure 2. A) Methylation status (Beta values) of *LTBP4* CpGs. including CpG island 102 (CGI 102) and CpG island 81 (CG1 81). in colon cancer (tumor) and paired normal colon samples (adjacent) from 96 colon cancer patients. and normal colon tissues from 39 healthy donors (NoCancer). Methylation difference is also included. as well as the CpG density and localization at genome level. B) Methylation levels and methylation difference of CGI 102. were all CpGs analized show statistically significant results when tumor is compared to normal colon tissue. C) Methylation levels and methylation difference between tumor, adjacent and normal tissue at CGI 81 were only two CpGs analyzed show statistically significant results. Further details on CpGs analyzed and p-values are included in Supplementary Table 2. Source: www.colonomics.org.

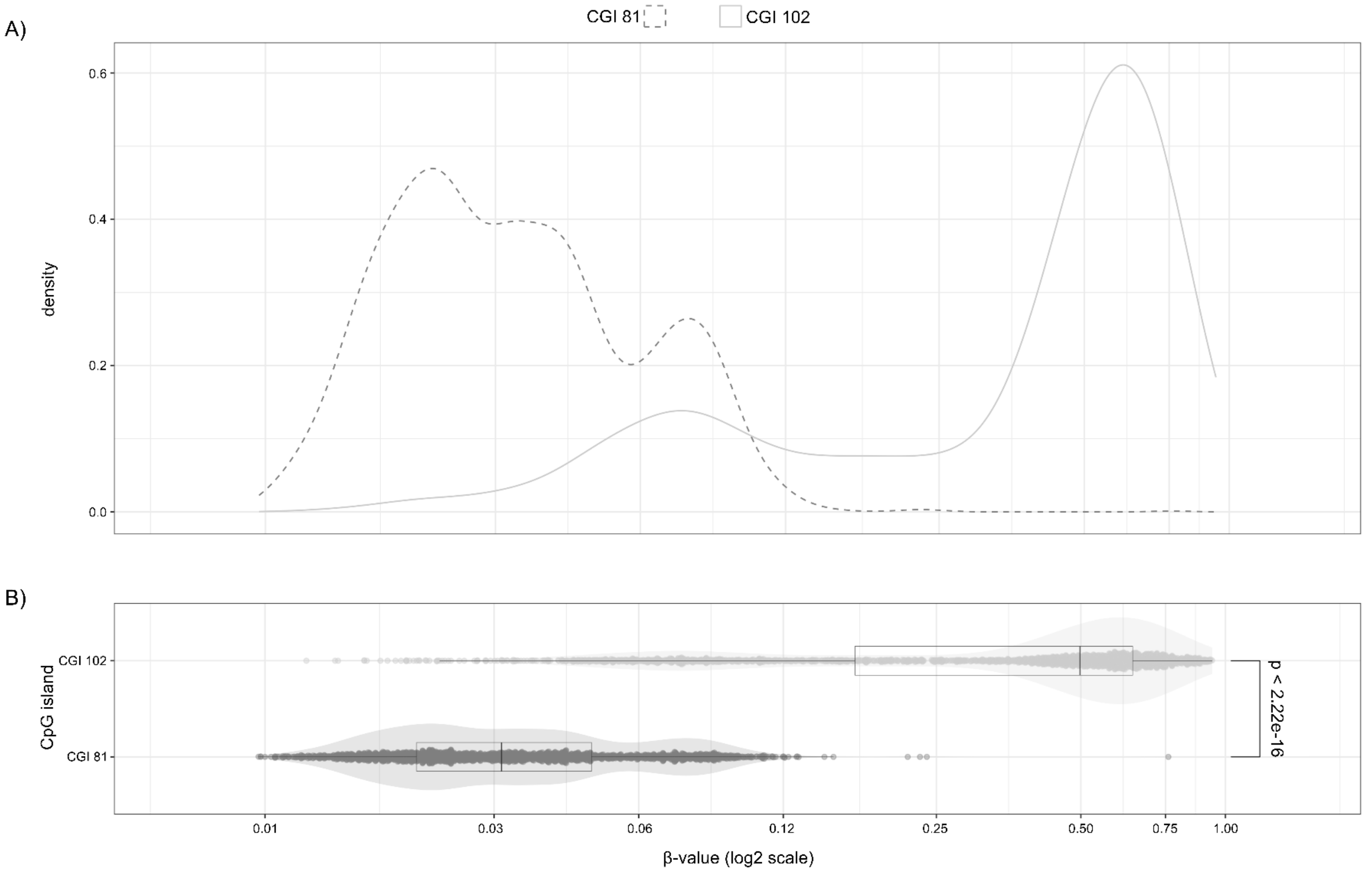

Supplementary Figure 3. Methylation profile (β-value in log2 scale) of CpG island (CGI) 81 and 102 of *LTBP4* in TCGA colorectal cancer cases (COADREA; n=736), showing a statistically significant methylation of CGI 102 compared to CGI 81. A) Density plot showing the distribution of the β-value mean of probes localized at CGI 81 (discontinuous line) and 102 (continuous line) for each sample. B) Boxplot representing β-values of probes localized at CGI 81 and 102 of all CRC cases. Source: https://xenabrowser.net/. Data type and experimental platform: Methylation β-values obtained using Illumina Human Methylation 450. CpGs at CGI 81 included: cg01000408 and cg18357908. CpGs at CGI102 included: cg06732228. cg03309253. cg27645259. cg11621464 and cg15768901.

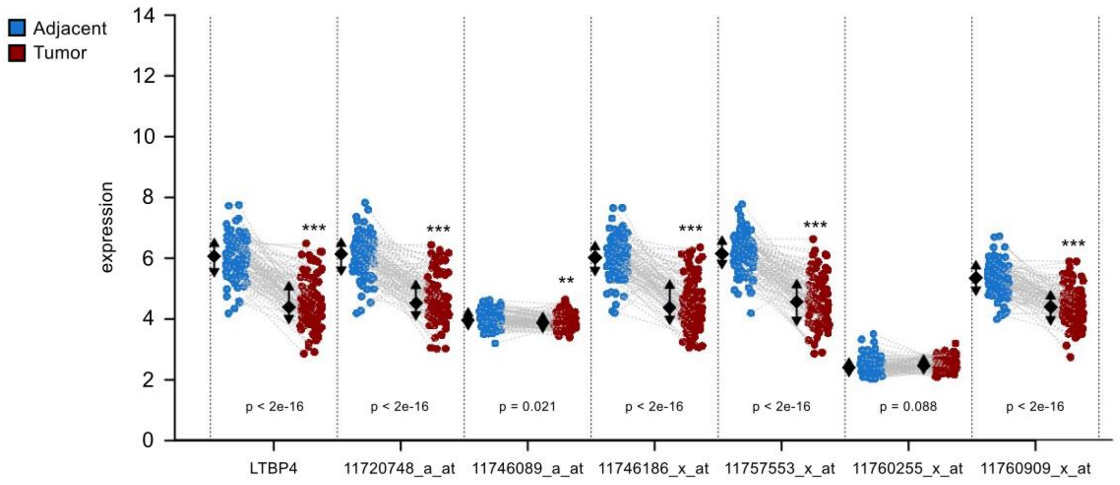

Supplementary Figure 4. Global mRNA expression levels of *LTBP4* of 98 colorectal tumor tissue compared to matched normal tissue. Expression levels of each single probe provided for the gene in the array are also included. Experimental platform: Affymetrix Human Genome U219 Array Plate platform. Source: www.colonomics.org.

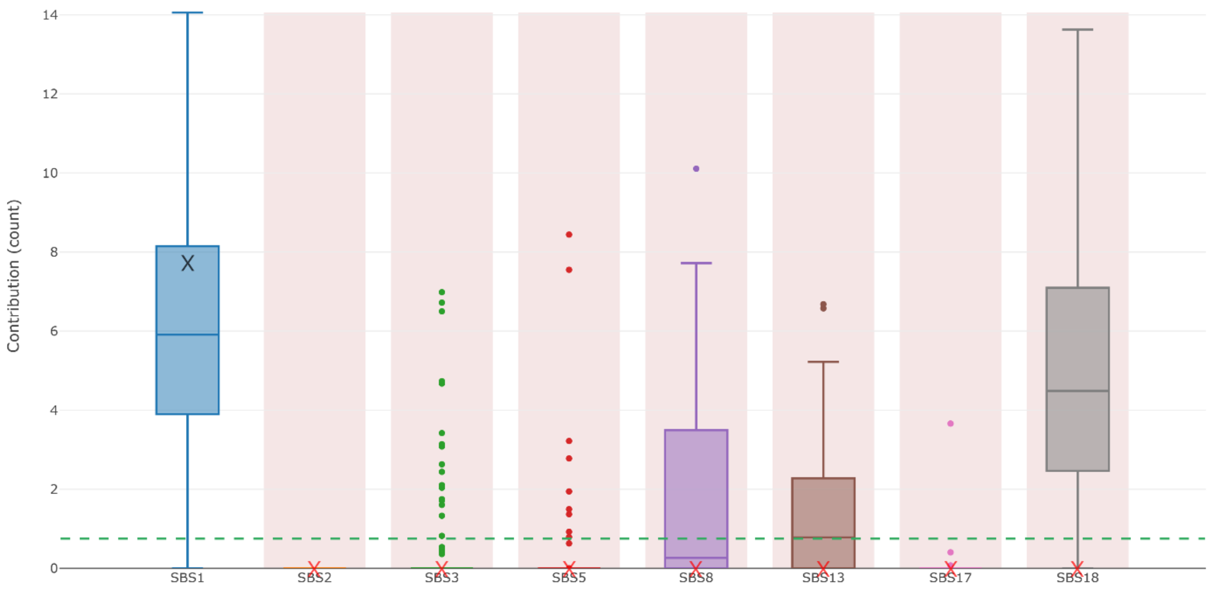

Supplementary Figure 5. Box plots with the contribution estimate distributions for each mutational signature of somatic variants identified in the tumor of patient P-8. The dashed green line represents the sparsity filter threshold. Source: Signal web application (https://signal.mutationalsignatures.com/).

REFERENCES

1. Schubert SA, Ruano D, Elsayed FA, et al. Evidence for genetic association between chromosome 1q loci and predisposition to colorectal neoplasia. *Br J Cancer*. 2017;118(2):e4.
2. Terradas M, Schubert SA, Viana-Errasti J, et al. Germline NPAT inactivating variants as cause of hereditary colorectal cancer. *Eur J Hum Genet*. Jul 2024;32(7):871-875. doi:10.1038/s41431-024-01625-8
3. Li H. Aligning sequence reads, clone sequences and assembly contigs with BWA-MEM. arXiv:1303.3997v2; 2013.
4. Díaz-Gay M, Vila-Casadesús M, Franch-Expósito S, Hernández-Illán E, Lozano JJ, Castellví-Bel S. Mutational Signatures in Cancer (MuSiCa): a web application to implement mutational signatures analysis in cancer samples. *BMC Bioinformatics*. 06 14 2018;19(1):224. doi:10.1186/s12859-018-2234-y
5. Degasperi A, Amarante TD, Czarnecki J, et al. A practical framework and online tool for mutational signature analyses show inter-tissue variation and driver dependencies. *Nat Cancer*. 02 2020;1(2):249-263. doi:10.1038/s43018-020-0027-5
6. Evans DGR, van Veen EM, Byers HJ, et al. A Dominantly Inherited 5' UTR Variant Causing Methylation-Associated Silencing of BRCA1 as a Cause of Breast and Ovarian Cancer. *Am J Hum Genet*. Aug 02 2018;103(2):213-220. doi:10.1016/j.ajhg.2018.07.002
7. Glodzik D, Bosch A, Hartman J, et al. Comprehensive molecular comparison of BRCA1 hypermethylated and BRCA1 mutated triple negative breast cancers. *Nat Commun*. Jul 27 2020;11(1):3747. doi:10.1038/s41467-020-17537-2
